# Investigating the Appeal of Nicotine Pouch Packaging, Flavour, and Nicotine Descriptors Among Adults in the UK: An Online Experiment

**DOI:** 10.1093/ntr/ntaf072

**Published:** 2025-03-26

**Authors:** Eve Taylor, Madeleine Ebdon, Matilda Nottage, Erikas Simonavicius, Leonie Brose, Ann McNeill, Deborah Arnott, Hazel Cheeseman, Laura Bunce, Katherine East

**Affiliations:** Department of Behavioural Science and Health, University College London, London, UK; Department of Addictions, King’s College London, London, UK; Department of Addictions, King’s College London, London, UK; Department of Addictions, King’s College London, London, UK; Department of Addictions, King’s College London, London, UK; Department of Addictions, King’s College London, London, UK; Department of Addictions, King’s College London, London, UK; Department of Behavioural Science and Health, University College London, London, UK; Action on Smoking and Health (ASH), London, UK; Action on Smoking and Health (ASH), London, UK; Action on Smoking and Health (ASH), London, UK; Department of Addictions, King’s College London, London, UK; Department of Primary Care and Public Health, Brighton and Sussex Medical School, Brighton, UK

## Abstract

**Introduction:**

Nicotine pouches have the potential to be used for tobacco harm reduction. Pouches can currently be sold in brightly coloured packaging with conceptual flavor and nicotine descriptors, which may appeal to youth. Therefore, the UK government are considering packaging regulations. We examined the impact of standardized packaging, limiting flavor names and standardizing nicotine descriptors on appeal and harm perceptions of nicotine pouches among adults.

**Methods:**

A 2024 Prolific Academic online experiment among UK Adults (*N* = 2,967) was used. Participants were randomized to one of four packaging conditions: (1) branded, (2) standardized with usual descriptors, (3) standardized with limited flavor descriptors, and (4) standardized with limited flavor and standardized nicotine descriptors. Logistic regressions examined associations between packaging conditions and (a) no interest in trying the products displayed and (b) harm perceptions relative to cigarettes.

**Results:**

There were no significant differences in reporting no interest in trying nicotine pouches in branded compared to standardized packaging with usual descriptors, standardized packaging with limited flavor descriptors, and standardized packaging with limited flavor and nicotine descriptors. When stratified by vaping/smoking status, participants who currently vaped had lower odds of reporting no interest in standardized packaging with limited flavor descriptors, compared to branded. There were no significant differences in harm perceptions by packaging condition.

**Conclusions:**

Overall, standardizing packaging, limiting flavor descriptors, and standardizing nicotine descriptors had little effect on adults’ interest in trying nicotine pouches or their perceptions of relative harm. There were, however, some differences by vaping status.

**Implications**

Findings suggest that if a standardized packaging, flavor, and nicotine descriptors policy is introduced for nicotine pouches to deter youth use, there might be little impact on appeal or harm perceptions among adults. This is important because nicotine pouches could be a helpful part of the toolkit for tobacco harm reduction.

## Introduction

Nicotine pouches are small pouches that can be put between the lip or gum to release nicotine into the bloodstream. They generally contain nicotine, flavors, sweeteners, and plant fiber, but exact ingredients vary between products.^1^ In 2020, current nicotine pouch use was low among 16–19-year-olds and adults in England, at 0.14% and 1.4% in 2020 respectively.^2,3^ However, ever use has since risen to 17% among adults who currently smoked, 7.2% among adults who used to smoke, and 1.5% among adults who had never smoked in 2024 (Brose et al; in press). Among 11–17-year-olds, 3.3% reported ever using nicotine pouches in 2024.^4^

There is little evidence of the overall health effects of nicotine pouches. Since nicotine pouches do not contain tobacco and do not involve combustion, they are likely less harmful than cigarettes, which kill up to two thirds of long-term users.^5^ Industry research has reported that nicotine pouches do not contain toxicants such as nitrosamines or polycyclic aromatic hydrocarbons,^6^ and toxicant exposure was reduced when people who smoked switched to nicotine pouch use for 7 days.^7^ Some nicotine pouches have been reported to contain Tobacco Specific Nitrosamines, however at substantially lower levels that cigarettes.^8^ Pharmacokinetic studies have also reported that nicotine pouches are effective at relieving nicotine cravings to a certain degree, so they may be an option for smoking cessation, although research here is very limited.^9^

In the UK, nicotine pouches do not currently have an age of sale limit, unlike cigarettes and vaping products (e-cigarettes and e-liquids) which cannot legally be sold to under 18s.^10^ Also unlike vaping products, nicotine pouches do not have a limit on their nicotine content, and the way nicotine strength is described is not standardized across brands. Similar to vapes, nicotine pouches are widely marketed,^11^ sold in brightly coloured packaging with a range of flavors,^12–14^ and evidence suggests these factors may increase their appeal.^15^ There is concern that the use of these products may cause nicotine dependence among youth. Therefore, in 2024, the UK government proposed new powers to regulate the marketing, packaging and flavors of all nicotine-containing products in the UK, including nicotine pouches.^16^ Canada has already introduced legislation restricting flavors, labeling, advertising, and limiting sales of nicotine pouches to behind the counter in pharmacies,^17^ but effects have not yet been evaluated.

Standardizing packaging and limiting flavor descriptors for cigarettes^18,19^ and vapes^20–22^ has been found to reduce their appeal among young people. However, standardizing packaging for vapes has also been found to inflate the inaccurate perception that vaping is equally/more harmful than smoking.^20^ Little is known about the effects of limiting flavor and standardizing nicotine descriptors for nicotine pouches.

As nicotine pouches have the potential to be used for tobacco harm reduction, it is important that any packaging regulations balance the need to discourage youth use, while not inflating inaccurate harm perceptions relative to cigarettes among adults who smoke. This study, therefore, aimed to examine associations between standardized nicotine pouch packaging, flavor descriptors and nicotine descriptors and (1) interest in trying nicotine pouches and (2) harm perceptions of nicotine pouches relative to cigarettes, among adults in the UK.

## Methods

### Participants

Data were from an online survey hosted on Qualtrics, collected between 24 May and 6 June 2024. Participants were adults aged 18 + years, drawn from Prolific Academic (a preexisting panel) with quota samples set to ensure the sample was representative of age, sex, and ethnicity in the UK.^23^ All participants provided informed consent. Ethical approval was granted by King’s College London (MRA-23/24-42484).

An initial sample of *N* = 3,025 was recruited, of whom 58 participants were removed due to missing demographic data, leaving an analytical sample of *N* = 2,967.

### Design

The design was based on previous studies conducted on standardized vape and cigarette packaging,^19–22,24^ where an experiment was embedded in an online survey. This design was chosen to investigate primarily standardized packaging, but also how the addition of extra regulation, such as standardizing flavor and nicotine descriptions could enhance the effect of standardized packaging. This was a between-subject experimental design where participants were randomly allocated at a 1:1:1:1 ratio to one of four conditions:

1. Branded packaging
2. Standardized packaging with usual descriptors
3. Standardized packaging with limited flavor descriptors
4. Standardized packaging with limited flavor descriptors and standardized nicotine strength descriptors

Each experimental condition included images of pouches from four different brands. Brands and flavors were chosen to represent the range of products currently available on the market.

Packaging images are available on the Open Science Framework https://osf.io/qpk8m.

### Measures

See https://osf.io/849nk for the full questionnaire.

#### Outcome Variables

Outcome measures were based on those used in previous research conducted on standardized vape and cigarette packaging.^19–22,24^

##### Interest in trying

Participants were shown a set of four images of nicotine pouch packs based on their experimental condition and asked, “Which of the following products would you be most interested in trying?” Participants could either select one of the four nicotine pouches, or response options “None of these products” or “I don’t know.” Responses were coded as “No Interest (None of these products)” vs “Interest (selecting any of the four products).” “I don’t know” responses represented less than 5% of the data (*n* = 42, 1.4%) and so they were removed from regression models.

##### Relative harm perceptions

Participants then viewed the “ZYN” branded pouch, based on the packaging condition (outlined above) they had previously been randomized to, and asked, “How harmful do you think it is to use this product?.” Response options included: “Not at all harmful,” “Harmful, but less harmful than smoking cigarettes,” “As harmful as smoking cigarettes,” “More harmful than smoking cigarettes,” and “Don’t Know.” In line with the limited research on nicotine pouch toxicant exposure^6^ and that pouches do not contain tobacco and do not involve combustion, perceptions were coded “Harmful, but less harmful than smoking cigarettes” or “Other (Not at all harmful, As harmful as smoking cigarettes, More harmful than smoking cigarettes, Don’t Know).”

#### Nicotine and Tobacco Use Measures

##### Nicotine pouch use

Participants were asked “Nicotine pouches are small pouches of nicotine which are placed in the mouth between your lip and gum. Brands include Lyft, Skruf, ZYN, Nordic Spirit, and Velo. Which of the following statements BEST applies to you?.” Response options included (1) I have tried nicotine pouches and still use them, (2) I have tried nicotine pouches but do not use them (anymore), (3) I have heard of nicotine pouches but have never tried them, (4) I have never heard of nicotine pouches, (5) Don’t know. Responses were coded as “Ever use (1-2)” and “Never use/Don’t know (3-5).”

##### Vaping status

Participants were asked “The following questions relate to vapes (also called e-cigarettes). Which of the following statements BEST applies to you?.” Response options included (1) I have never tried vapes (e-cigarettes), (2) I have tried vapes (e-cigarettes) but do not use them (anymore), (3) I have tried vapes (e-cigarettes) and still use them. Responses were coded: “Currently vaping” (3), “Past vaping” (2), “Never vaping” (1).

##### Smoking status

Participants were asked “Which of the following best applies to you? Please note we are referring to cigarettes and other kinds of tobacco that you set light to and NOT “heated tobacco (eg, IQOS).” Response options included (1) I smoke cigarettes (including hand-rolled) every day, (2) I smoke cigarettes (including hand-rolled), but not every day, (3) I do not smoke cigarettes at all, but I do smoke tobacco of some kind (eg, Pipe, cigar or shisha), (4) I have stopped smoking completely in the last year, (5) I stopped smoking completely more than a year ago, (6) I have never been a smoker (ie, smoked for a year or more). Responses were coded: “Currently smoking” (1-3), “Past smoking” (4-5), “Never smoking” (6).

#### Demographic Measures

Exact measures are outlined in Supplementary File 1.

Age was coded “18-24,” “25-34,” “35-44,” “45-54,” “55 or older.”

Gender was recorded as “Male,” “Female,” “In another way.” For regression analyses, gender was coded “Female” and “Other” (male or in another way) due to small sample sizes for. “Prefer not to say” responses (*n* = 7) were removed.

Perceived Financial Status (PFS) was coded as “Comfortable on present income,” “Coping on present income,” “Finding it difficult on present income,” “Finding it very difficult on present income,” “Don’t know.” Prefer not to say responses (*n* = 40) were removed.

Ethnicity was coded (1) Asian, Asian British, (2) Black, Black British, Caribbean or African, (3) Mixed or Multiple ethnic groups, (4) White, (5) Other ethnic group, in line with the Census definitions.^25^ Due to small sample sizes for regression analysis, ethnicity was coded “White (4),” and “Racialised minorities (1-3, 5).”’Prefer not to say’ responses (*n* = 15) were removed.

### Data Analysis

Analyses were pre-registered on the Open Science framework: https://osf.io/849nk.

Frequencies were used to describe current nicotine pouch use, vaping and smoking status and participant demographics. To test for successful randomization, chi-square tests were used to test for differences in participant demographics by condition.

To investigate research question 1, logistic regressions examined associations between “No interest” in trying any of the displayed nicotine pouches and condition (branded packaging vs the three standardized packaging conditions). Analysis was first unadjusted, and then adjusted for pouch use, smoking status, vaping status, PFS, age, gender, and ethnicity.

To investigate research question 2, logistic regressions examined associations between nicotine pouch harm perceptions relative to smoking and nicotine pouch packaging condition (branded packaging vs the three standardized packaging conditions). Analyses were first run unadjusted, and then adjusted for nicotine pouch use, smoking status, vaping status, perceived financial status, age, gender, and ethnicity.

#### Deviation from the Pre-registered Analyses

Due to the small proportion of “Don’t Know” responses across all packaging conditions for interest in trying (*n* = 42, 1.4%), planned sensitivity analyses using multinomial logistic regressions comparing “Interest (reference),” “No interest” and “Don’t know” were not conducted.

In addition to the pre-registered analyses, to further investigate differences in interest in trying by smoking and vaping status, and relative harm perceptions by smoking status, exploratory analyses were conducted by repeating logistic regression analyses stratifying participants by smoking and vaping status.

### Sensitivity Analyses

First, to explore the different relative harm perceptions, logistic regressions examined associations between each of the nicotine pouch harm perceptions vs all other perceptions and nicotine pouch packaging condition. Analyses were adjusted for nicotine pouch use, smoking status, vaping status, perceived financial status, age, gender, and ethnicity.

Second, as people who have never heard of pouches would not know how harmful products are, analyses were also repeated excluding people who had never heard of, or do not know if they have heard of nicotine pouches.

## Results

### Sample Characteristics

Half of participants were female (51.5%), most identified as white (86.0%), and perceived their financial status as “coping” (47.8%) or “comfortable” (26.6%). Few participants had used nicotine pouches (13.0%), with most having heard of but never tried pouches (58.2%), just under a third had never heard of pouches (28.4%), and few did not know if they had tried nicotine pouches (0.4%). Just under a fifth of participants currently smoked (19.3%) and/or currently vaped (17.1%) (Table 1).

**Table 1.** Participant Characteristics of Nicotine Pouch Study; Adults in the UK 2024 (*N* = 2967)

	Total	Branded packaging	Standardized packaging with usual descriptors	Standardized packaging with limited flavor descriptors	Standardized with limited flavor and standardized nicotine descriptors
	%(*n*)	%(*n*)	%(*n*)	%(*n*)	%(*n*)
Total	100(2967)	25.7(762)	24.0(713)	24.5(726)	25.8(766)
**Gender**					
Female	51.5(1527)	51.0(389)	51.9(370)	52.9(384)	50.1(384)
Male	48.0(1425)	48.5(361)	47.5(339)	46.6(338)	49.5(379)
Identify in another way	0.5(15)	0.5(4)	0.6(4)	0.6(4)	0.4(3)
**Perceived financial status**					
Comfortable	26.6(790)	26.5(202)	26.4(188)	26.9(195)	26.8(205)
Coping	47.8(1419)	47.1(359)	49.4(352)	47.0(341)	47.9(367)
Finding it difficult	18.0(534)	18.2(139)	17.4(124)	18.3(133)	18.0(138)
Finding it very difficult	7.6(224)	8.2(62)	6.9(49)	7.9(57)	7.3(56)
**Age**					
18–24	10.6(314)	10.4(79)	9.4(67)	10.6(77)	11.9(91)
25–34	17.2(511)	16.9(129)	16.1(115)	17.4(126)	18.4(141)
35–44	16.3(483)	16.8(128)	16.4(117)	15.8(115)	16.1(123)
45–54	17.2(510)	17.2(131)	17.0(121)	17.8(129)	16.8(129)
55 or older	38.7(1149)	38.7(295)	41.1(293)	38.4(279)	36.8(282)
**Country**					
England	84.6(2512)	86.6(661)	82.6(589)	84.3(612)	84.9(650)
Scotland	4.4(131)	3.9(30)	5.5(39)	4.4(32)	3.9(30)
Wales	8.4(249)	7.7(59)	8.7(62)	8.1(59)	9.0(69)
Northern Ireland	2.6(76)	1.7(13)	3.2(23)	3.2(23)	2.2(17)
**Ethnicity**					
Asian, Asian British	7.8(232)	8.1(62)	8.0(57)	7.6(55)	7.6(58)
Black, Black British, Caribbean or African	3.6(106)	3.7(28)	3.1(22)	3.2(23)	4.3(33)
Mixed or Multiple Ethnic groups	1.4(43)	0.9(7)	1.4(10)	1.7(12)	1.8(14)
White	86.0(2553)	86.4(659)	86.1(614)	86.1(625)	85.4(655)
Other Ethnic Group	1.1(33)	0.8(6)	1.4(10)	1.5(11)	0.8(6)
**Pouch status**					
Not used/ Don’t know	87.0(2584)	88.1(672)	88.2(629)	87.9(638)	84.1(645)
Ever used	13.0(385)	11.9(91)	11.8(84)	12.1(88)	15.9(122)
**Vaping status**					
Currently vape	17.1(506)	15.2(115)	19.1(136)	17.8(129)	16.4(126)
Used to vape	24.8(737)	24.0(183)	22.2(158)	26.3(191)	26.9(206)
Never vaped	58.1(1724)	60.9(464)	58.8(419)	55.9(406)	56.7(435)
**Smoking status**					
Currently smoke	19.3(573)	18.9(144)	20.1(143)	18.6(135)	19.7(151)
Used to smoke	30.6(909)	29.9(228)	29.3(209)	33.3(242)	30.0(230)
Never smoked	50.1(1485)	51.2(390)	50.6(361)	48.1(349)	50.3(386)

### Interest in Trying Nicotine Pouches

Across all conditions, 27.0% of participants reported interest in trying a nicotine pouch and 73.0% reported no interest. In unadjusted and adjusted analyses, there was no significant difference in participants reporting no interest in trying nicotine pouches in branded packs (75.1%) compared to standardized packaging with usual descriptors (70.6%), standardized packaging with limited flavor descriptors (72.1%) and standardized packaging with limited flavor and nicotine descriptors (74.1%) (Table 2, Supplementary Figure 1).

**Table 2.** Associations Between Interest in Trying Nicotine Pouches, Packaging Condition and Participant Characteristics; Adults in the UK, 2024 (*N* = 2,967) ^a^

	Interest in trying (ref)^a^	No interest in trying ^b^			Harmful, but less harmful than smoking cigarettes (ref)^c^	Other perception ^d^		
	%(*n*)	%(*n*)	AOR(95%CI)	*p*	%(*n*)	%(*n*)	AOR(95%CI)	*p*
**Total**	27.0(789)	73.0(2136)			57.1(1693)	42.9(1274)		
**Packaging condition**								
Branded packaging	24.9(186)	75.1(562)	1	ref	55.4(422)	44.6(340)	1	ref
Standardized packaging with usual descriptors	29.4(207)	70.6(498)	0.76(0.57-1.01)	.056	58.5(417)	41.5(296)	0.87(0.70-1.08)	.207
Standardized packaging with limited flavor descriptors	27.9(200)	72.1(516)	0.92(0.69-1.23)	.582	57.9(420)	42.1(306)	0.92(0.74-1.14)	.447
Standardized with limited flavor and standardized nicotine descriptors	25.9(196)	74.1(560)	1.15(0.87-1.54)	.329	56.7(434)	43.3(332)	1.00(0.81-1.23)	.999
**Gender+**								
Other ^c^	30.9(435)	69.1(975)	1	ref	63.2(910)	36.6(530)	1	ref
Female	23.4(354)	76.6(1161)	0.97(0.79–1.19)	.779	51.3(783)	48.7(744)	1.50(1.29–1.75)	**<.001**
**Ethnicity+**								
White	25.9(651)	74.1(1866)	1	ref	58.2(1486)	41.8(1067)	1	ref
Racialized minorities ^c^	33.9(138)	66.2(270)	0.77(0.58–1.02)	.070	50.0(207)	50.0(207)	1.65(1.32–2.07)	**<.001**
**Age (years)**								
18–24	42.7(132)	57.3(177)	1	ref	71.3(224)	28.7(90)	1	ref
25–34	39.8(202)	60.2(305)	1.19(0.83–1.69)	.346	67.7(346)	32.3(165)	1.16(0.85–1.59)	.353
35–44	36.1(172)	63.9(304)	1.36(0.93–1.97)	.112	56.5(273)	43.5(210)	1.83(1.33–2.51)	**<.001**
45–54	23.6(118)	76.4(382)	2.98(2.00–4.43)	**<.001**	56.1(286)	43.9(224)	1.84(1.34–2.53)	**<.001**
55 or older	14.6(165)	85.4(968)	4.23(2.91–6.15)	**<.001**	49.1(564)	50.9(585)	2.44(1.81–3.29)	**<.001**
**Perceived Financial Status**								
Comfortable	21.8(171)	78.2(614)	1	ref	54.7(432)	45.3(358)	1	ref
Coping	26.9(376)	73.1(1021)	1.00(0.77–1.29)	.980	57.6(818)	42.4(601)	0.94(0.78–1.12)	.473
Finding it difficult	32.1(169)	67.9(358)	0.98(0.72–1.34)	.890	60.7(324)	39.3(210)	0.88(0.69–1.11)	.273
Finding it very difficult	33.8(73)	66.2(143)	0.86(0.57–1.30)	.467	53.1(119)	46.9(105)	1.21(0.89–1.55)	.229
**Pouch status**								
Never used/ Don’t know	21.1(538)	78.9(2012)	1	ref	54.6(1411)	45.4(1172)	1	ref
Ever used	67.0(251)	33.0(124)	0.39(0.30–0.52)	**<.001**	73.4(282)	26.6(102)	0.62(0.48–0.82)	**<.001**
**Vaping status**								
Currently vape	65.1(321)	24.9(172)	1	ref	71.1(360)	28.9(146)	1	ref
Used to vape	44.4(321)	55.6(402)	1.73(1.33–2.26)	**<.001**	63.8(470)	36.2(267)	1.51(1.16–1.96)	**.002**
Never vaped	8.6(147)	91.4(1562)	6.38(4.67–8.72)	**<.001**	50.1(863)	49.9(861)	2.11(1.60–2.78)	**<.001**
**Smoking status**								
Currently smoke	63.7(353)	36.3(202)	1	ref	60.4(346)	39.6(227)	1	ref
Used to smoke	28.6(256)	71.4(639)	2.40(1.85–3.12)	**<.001**	60.3(548)	39.7(361)	0.67(0.53–0.85)	**.001**
Never smoked	12.2(180)	87.8(1295)	4.83(3.52–6.63)	**<.001**	53.8(799)	46.2(686)	0.72(0.56–0.94)	**.015**

^a^models are adjusted for vaping status, pouch use, age, gender, ethnicity and perceived financial status.

^b^Don’t know responses (*N* = 42, 1.4%) were removed from “interest” analysis, in line with the pre-registration (if < 5% don’t know this response is excluded from analyses).

^c^Due to small cell counts, ethnicity was collapsed into “White” and “ Racialised minorities.” Gender was also collapsed into “Female” and “Other.”.

^d^“Other perceptions” include: “not at all harmful,” “as harmful as smoking cigarettes,” “more harmful than smoking cigarettes,” “don’t know.”.

When the three standardized packs were compared, participants randomized to view standardized packaging with limited flavor and standardized nicotine descriptors had higher odds of reporting no interest compared to standardized packaging with usual descriptors (74.1% vs 70.6%; AOR = 1.53 95%CI = 1.14–2.04, *p* = .004). There were no other significant differences in interest between the different types of standardized packs.

Age was the only participant demographic that was significantly associated with interest in trying. Across all packaging conditions, participants who were aged 44–55 years (76.4%) or 55 or older (85.4%) had greater odds of reporting no interest compared to participants aged 18–24 (57.3%) (Table 2).

Irrespective of packaging condition, participants who used to vape (55.6%) or had never vaped (91.4%) had significantly greater odds of reporting no interest in trying pouches than participants who currently vaped (24.9%). Participants who used to smoke (71.4%) or had never smoked (87.8%) also had greater odds of reporting no interest in trying pouches compared to participants who currently smoked (36.3%) (Table 2).

When analyses were stratified by smoking status, there were no significant differences in interest in trying by packaging condition (Table 3).

**Table 3.** Associations Between Interest in Trying, Harm Perceptions, and Nicotine Pouches and Packaging Condition, Stratified by Smoking and Vaping Status; Adults in the UK 2024 (*N* = 2967)^a^

	Interest in trying (ref)^c^	No interest in trying^c^			Harmful, but less harmful than smoking cigarettes (ref)	Other ^b^		
	%(*n*)	%(*n*)	AOR(95%CI)	*p*	%(*n*)	%(*n*)	AOR(95%CI)	*p*
**Currently smoking**	(*n* = 555)^c^				(*n* = 573)			
Branded packaging	60.3(82)	39.7(54)	1	ref	63.9(92)	36.1(52)	1	ref
Standardized packaging with usual descriptors	65.9(91)	34.1(47)	0.79(0.49–1.30)	.357	58.0(83)	42.0(60)	1.33(0.81–2.18)	.262
Standardized packaging with limited flavor descriptors	65.9(87)	34.1(45)	0.80(0.48–1.31)	.366	56.3(76)	43.7(59)	1.37(0.83–2.28)	.217
Standardized packaging with limited flavor and standardized nicotine descriptors	62.4(93)	37.6(56)	0.93(0.58–1.49)	.750	62.9(95)	37.1(56)	1.10(0.67–1.81)	.702
**Used to smoke**	(n = 895)^c^				(N = 909)			
Branded packaging	27.2(61)	72.8(163)	1	ref	60.1(137)	39.9(91)	1	ref
Standardized packaging with usual descriptors	32.2(67)	67.8(141)	0.79(0.52–1.19)	.258	61.2(128)	38.8(81)	0.94(0.63–1.40)	.756
Standardized packaging with limited flavor descriptors	30.0(71)	70.0(166)	0.88(0.58–1.31)	.518	62.4(151)	37.6(91)	0.91(0.62–1.34)	.622
Standardized packaging with limited flavor and standardized nicotine descriptors	25.2(57)	74.8(169)	1.11(0.73–1.69)	.628	57.4(132)	42.6(98)	1.16(0.79–1.71)	.459
**Never smoked**	(*n* = 1475)^c^				(*n* = 1485)			
Branded packaging	11.1(43)	88.9(345)	1	ref	49.5(193)	50.5(197)	1	ref
Standardized packaging with usual descriptors	13.6(49)	86.4(310)	0.79(0.51–1.22)	.287	57.1(206)	42.9(155)	0.71(0.52–0.96)	**.025**
Standardized packaging with limited flavor descriptors	12.1(42)	87.9(305)	0.91(0.58–1.42)	.666	55.3(193)	44.7(156)	0.78(0.57–1.05)	.102
Standardized packaging with limited flavor and standardized nicotine descriptors	12.1(46)	87.9(335)	0.91(0.73–1.42)	.677	53.8(207)	46.2(178)	0.88(0.65–1.18)	.388

^a^models are adjusted for vaping status, pouch use, age, gender, ethnicity and perceived financial status.

^b^“Other perceptions” include: “not at all harmful,” “as harmful as smoking cigarettes,” “more harmful than smoking cigarettes,” “don’t know.”.

^c^Don’t know responses (N = 42, 1.4%) were removed from analysis, in line with the pre-registration (if < 5% don’t know this response is excluded from analyses).

Due to small cell counts, ethnicity was collapsed into “White” and “Racialised minorities.” Gender was also collapsed into “Female” and “Other.”.

When analyses were stratified by vaping status, participants who currently vaped had lower odds of reporting no interest in trying pouches in standardized packaging with limited flavor descriptors (28.3%) compared to branded packs (41.3%). There were no significant differences for any other vaping status group (Supplementary Table 1).

### Relative Harm Perceptions

Across all packaging conditions, 3.3% of participants perceived pouches as “not at all harmful,” 57.1% as “harmful, but less harmful than smoking cigarettes,” 24.9% as “equally harmful as smoking cigarettes,” 5.2%, as “more harmful than smoking cigarettes,” and 9.5% reported they did not know.

When exploring effects of standardized packaging on harm perceptions, in both unadjusted and adjusted analyses, there was no significant difference by packaging condition (Table 2). There were also no significant differences in harm perceptions between the three types of standardized packs (*p* > .05).

Irrespective of packaging conditions, perceptions that nicotine pouches are “harmful, but less harmful than smoking cigarettes” were significantly lower among participants who identified as female compared to another gender; participants who identified as a racialized minority compared to white; and participants who were 55+, 45–55, or 35–44 compared to 18–24 (Table 2). Across all packaging conditions, those who currently vaped were more likely to perceive that the product displayed is harmful, but less harmful than smoking cigarettes than those who used to vape or had never vaped (Table 2).

When analyses were stratified by smoking status, participants who had never smoked were more likely to perceive the nicotine pouches “harmful, but less harmful than smoking cigarettes” when products were in standardized packs with usual descriptors (57.1%) compared to branded packs (49.5%) (Table 3). There were no other significant differences (Table 3).

### Sensitivity Analyses

When exploring effects of standardized packaging on each of the five relative harm perceptions, there were no significant differences in reporting each of relative harm perceptions between the three types of standardized packs (*p* > .05) (Supplementary Table 2).

When people who had never heard of or did not know if they had heard of nicotine pouches were removed, there was no significant difference in harm perceptions by packaging condition (Supplementary Table 3).

## Discussion

There was little difference in UK adults reporting interest in trying nicotine pouches between products presented in standardized or branded packaging. However, when looking at only the three standardized packaging conditions, interest in trying was significantly greater for nicotine pouches in standardized packaging with usual descriptors compared to standardized packaging with limited flavor and standardized nicotine descriptors. Standardizing packaging also had little significant effect overall on the relative harm perceptions of nicotine pouches, although there were some differences by smoking status: incorrect harm perceptions were more common among people who had never smoked and viewed standardized packs with usual descriptors, compared with branded packs.

The findings that standardizing nicotine pouch packaging had little impact on adults’ interest in trying them are in line with previous research on standardized vape packaging among adults.^22^ However, limiting flavor and standardizing nicotine descriptors did reduce interest in trying when nicotine pouch packaging was standardized, consistent with previous research on e-liquid flavors among youth.^21^ Due to the experimental design, we were unable to determine the effects of limiting flavor and standardizing nicotine descriptors in isolation from the effects of standardized packaging. However, in relation to the UKs tobacco and nicotine products regulatory policy, it is unlikely that new regulations would target nicotine and/or flavor descriptors only without standardizing nicotine pouch packaging.

Similar to misperceptions of vapes,^26^ harm perceptions that nicotine pouches were more or equally as harmful as cigarettes were common, with 30% of participants considering the pouches that they were shown to be equally or more harmful than cigarettes. This may be due to overall inaccurate harm perceptions of the role that nicotine plays in the health effects of tobacco smoking.^27,28^ Also, almost a third of participants had never heard of pouches (28.4%), therefore those unfamiliar with them may have been unsure about their relative harms. Contrary to findings assessing vape packaging among youth,^20^ standardizing packaging in this study did not change harm perceptions among adults. However, similarly to previous research on nicotine pouches, which found little difference in harm perceptions between pouches with conceptual flavor names (dark frost) and characterizing names (coffee), we found little effect of flavor descriptors on any of the relative harm perceptions.^29^

The combination of findings, suggesting that there is little impact of standardizing nicotine pouch packaging on interest in trying and harm perceptions, is promising. Overall they suggest that if the UK government does introduce standardized packaging for nicotine pouches to deter youth use^16^ there might be little impact among adults. This is important because nicotine pouches could potentially be part of the toolkit for tobacco harm reduction. The evidence for standardizing flavor descriptors is less clear, although this should still be considered as our previous research on vaping products has suggested this may reduce their appeal to youth.^21^ Standardizing nicotine descriptors might also provide consumers with more accurate information on their nicotine use. This is particularly important because there is often confusion around the strength of nicotine described as mg/ml or as a percentage in vapes^30^; therefore, a standardized scale for conveying nicotine strength should be considered across nicotine products.^31^ Age of sale should also be introduced across all nicotine products, as youth under the age of 18 can currently legally purchase nicotine pouches. If packaging regulations are introduced for nicotine pouches, policymakers should be mindful of new products that utilize loopholes in regulation, as was seen with the introduction of limited-edition cigarette tins during the phase-in of standardized cigarette packaging and the introduction of short-fill e-liquids in response to caps on e-liquid concentration and bottle size.^10,32^

This study has several strengths. First, this study was based on previous work using similar designs, using a randomized experiment to reduce confounding.^20–22^ Second, it was the first study of its kind to assess the impact of standardizing nicotine pouch packaging among adults, and it is timely for UK policy.^16^

There are also several limitations. We used quota sampling to achieve a sample nationally representative of age, sex, and ethnicity. Although our sample was broadly in line with 2021 Census data for age, sex, and ethnicity, it was skewed toward people with higher socioeconomic status and current smoking, vaping and nicotine pouch use were higher than estimates from national surveys^26,33^; therefore, the sample may not be truly representative in terms of the use of nicotine products in the UK population. Second, it is uncertain whether findings on interest in the use of nicotine pouches would translate into purchasing and actual use, particularly because of the lack of ecological validity from online experiments compared to the real-world retail environment. Third, harm perceptions questions were only based on one of the brand images (ZYN), therefore, findings may not be generalized to other brands, especially brands with different styles of packaging. Fourth, the sample was among adults only and future research should explore the effects of standardizing packaging among youth, who should be deterred from using nicotine pouches especially due to regulatory loopholes meaning that under 18s can legally purchase them in the UK.

## Conclusions

There was little evidence of an effect of standardizing packaging and limiting flavor and standardizing nicotine descriptors on UK adults’ interest in trying nicotine pouches or harm perceptions relative to smoking.

## Supplementary Material

ntaf072_suppl_Supplementary_Materials

## Data Availability

Data are available on a reasonable request to eve.taylor@ucl.ac.uk
