# Supplementary material for: Investigating the Appeal of Nicotine Pouch Packaging, Flavour, and Nicotine Descriptors Among Adults in the UK: An Online Experiment
Source: Nicotine Tob Res. 2025 Mar 26;28(2):251–9. doi: 10.1093/ntr/ntaf072 (PMC12824943; doi:10.1093/ntr/ntaf072)
Supplement: ntaf072_suppl_Supplementary_Materials [file ntaf072_suppl_supplementary_materials.docx]

## Prolific Academic quota sampling measures

The following measures are used by prolific academic to provide quota sampling. Participants are asked these questions when they sign up to the prolific academic platform (1).

**Age**: What is your date of birth?

**Sex:** What is your sex, as recorded on legal/official documents?

Male

Female

**Ethnicity:** What ethnic group do you belong to?

Asian

Black

Mixed

White

Other

Prefer not to say

## Survey demographic measures

The following measures were designed by the researchers to capture participant demographics

***Age:*** Participants were asked “How old are you?” With the following response options:

18-24

25-34

35-44

45-54

55+

***Gender****:* Participants were asked “Which of the following describes how you think of yourself?”. With the following response options:

1. Male

2. Female

3. In another way

4. Prefer not to say

Gender was recorded as ‘Male’, ‘Female’, ‘In another way’. For regression analyses, gender was coded ‘Female’ and ‘Other’ (male or in another way) due to small sample sizes for. ‘Prefer not to say’ responses (n=7) were removed. This question was informed by the National Centre for Social Research (2).

***Perceived financial stress (PFS):*** Participants were asked “Which, if any, of the below statements best defines your attitude towards your present income?” with the following response options:

- Comfortable on present income
- Coping on present income
- Finding it difficult on present income
- Finding it very difficult on present income
- Don’t know
- Prefer not to say

Perceived Financial Status (PFS) was coded as ‘Comfortable on present income’, ‘Coping on present income’, ‘Finding it difficult on present income’, ‘Finding it very difficult on present income’, ‘Don’t know’. Prefer not to say responses (n=40) were removed.

***Ethnicity:*** Participants were asked “What ethnic group best describes you? Please select one option only. (We ask the question in this way so that it is consistent with Census definitions.)” with the following response options:

1. Asian, Asian British
2. Black, Black British, Caribbean or African
3. Mixed or multiple ethnic groups
4. White
5. Other ethnic group

Consistent with prior work (3), Due to small sample sizes for regression analysis, ethnicity was coded ‘White (4)’, and ‘Racialised minorities (1-3, 5)’. ’Prefer not to say’ responses (n=15) were removed.

Alt text: The graph shows the percentage of adults reporting interest in trying nicotine pouches by packaging condition. The bars are highest for 'no interest', ranging from 70.6%-75.1% depending on the packing condition. The bars are lower for 'interest in trying' ranging from 24.9%-29.4% depending on the packaging condition. The darkest green bars represent people who saw branded packaging, the second darkest green bars represent people who saw standardised packaging with usual descriptions, the third darkest green bars represent people who saw standardised packaging with standardised flavour descriptions, and the lightest green bars represent people who saw standardised packaging with standardised flavour and nicotine descriptions.

| Supplementary table 1: Associations between interest in trying nicotine pouches and packaging condition, stratified by vaping status; Adults in the UK 2024 (N=2925) | | | | |
| --- | --- | --- | --- | --- |
|  | Interest in trying (ref) | No interest in trying | | |
|  | %(n) | %(n) | AOR(95%CI) | p |
| **Currently vaping (n=493)a** |  |  |  |  |
| Branded packaging | 58.7(64) | 41.3(45) | 1 | ref |
| Standardised packaging with usual descriptors | 65.2(86) | 34.8(46) | 0.77(0.46-1.30) | .333 |
| Standardised packaging with limited flavour descriptors | 71.7(91) | 28.3(36) | 0.57(0.33-0.98) | **.043** |
| Standardised packaging with limited flavour and standardised nicotine descriptors | 64.0(80) | 36.0(45) | 0.81(0.48-1.38) | .440 |
| **Used to vape (n=723)a** |  |  |  |  |
| Branded packaging | 44.4(80) | 55.6(100) | 1 | ref |
| Standardised packaging with usual descriptors | 50.0(78) | 50.0(78) | 0.80(0.52-1.23) | .309 |
| Standardised packaging with limited flavour descriptors | 43.3(81) | 56.7(106) | 1.05(0.69-1.58) | .828 |
| Standardised packaging with limited flavour and standardised nicotine descriptors | 40.0(82) | 59.0(119) | 1.16(0.77-1.74) | .472 |
| **Never vaped (n=1709)a** |  |  |  |  |
| Branded packaging | 9.2(42) | 90.8(417) | 1 | ref |
| Standardised packaging with usual descriptors | 10.3(43) | 89.7(374) | 0.88(0.56-1.37) | .562 |
| Standardised packaging with limited flavour descriptors | 7.0(28) | 93.0(374) | 1.35(0.82-2.21) | .243 |
| Standardised packaging with limited flavour and standardised nicotine descriptors | 7.9(34) | 92.1(397) | 1.18(0.73-1.89) | .501 |
| a models are adjusted for smoking status, pouch use, age, gender, ethnicity and perceived financial status  Due to small cell counts, ethnicity was collapsed into ‘White’ and ‘Racialised minorities’. Gender was also collapsed into ‘Female’ and ‘Other’.  Don’t know responses (N=42, 1.4%) were removed from analysis, in line with the pre-registration (if <5% don’t know this response is excluded from analyses). | | | | |

| Supplementary table 2: Associations between harm perceptions of nicotine pouches and packaging condition; Adults in the UK, 2024 (N=2,967) | | | | | | | | | | | | | | | | |
| --- | --- | --- | --- | --- | --- | --- | --- | --- | --- | --- | --- | --- | --- | --- | --- | --- |
|  | Not at all harmful  vs  All other perceptions ^a^ | | | Harmful, but less harmful than smoking cigarettes  vs  All other perceptions ^b^ | | | As harmful as smoking cigarettes  vs  All other perceptions ^c^ | | | More harmful than smoking cigarettes  Vs  All other perceptions ^d^ | | | Don't know  Vs  All other perceptions ^e^ | | |  |
|  | %(n) | AOR  (95% CI) | p | %(n) | AOR  (95% CI) | p | %(n) | AOR  (95% CI) | p | %(n) | AOR  (95% CI) | p | %(n) | AOR  (95% CI) | p |  |
| Banded | 2.6(20) | 1 | ref | 55.4(422) | 1 | ref | 26.4(201) | 1 | ref | 4.7(36) | 1 | ref | 10.9(83) | 1 | ref |  |
| Standardised packaging with usual descriptors | 3.9(28) | 0.69  (0.38-1.24) | .211 | 58.5(417) | 0.87  (0.70-1.08) | .207 | 22.3(159) | 1.26  (0.99-1.61) | .062 | 6.5(46) | 0.71  (0.45-1.11) | .131 | 8.8(63) | 1.26  (0.89-1.78) | .197 |  |
| Standardised packaging with limited flavour descriptors | 4.0(29) | 0.67  (0.37-1.20) | .177 | 57.9(420) | 0.92  (0.74-1.14) | .447 | 24.0(174) | 1.12  (0.88-1.43) | .348 | 5.4(39) | 0.84  (0.53-1.34) | .468 | 8.8(64) | 1.25  (0.88-1.76) | .217 |  |
| Standardised with limited flavour and standardised nicotine descriptors | 2.9(22) | 0.95  (0.52-1.77) | .873 | 56.7(434) | 1.00  (0.81-1.23) | .999 | 26.8(205) | 0.93  (0.74-1.18) | .566 | 4.4(34) | 1.03  (0.64-1.67) | .903 | 9.3(71) | 1.14  (0.81-1.60) | .441 |  |
| Models are adjusted for vaping status, pouch use, age, gender, ethnicity and perceived financial status  Due to small cell counts, ethnicity was collapsed into ‘White’ and ‘Racialised minorities’. Gender was also collapsed into ‘Female’ and ‘Other’  a‘Other perceptions’ include, ‘harmful, but less harmful than smoking cigarettes’, ‘as harmful as smoking cigarettes’, ‘more harmful than smoking cigarettes’, ‘don't know’.  b‘Other perceptions’ include ‘not at all harmful’, ‘as harmful as smoking cigarettes’, ‘more harmful than smoking cigarettes’, ‘don't know’.  c‘Other perceptions’ include ‘not at all harmful’, ‘harmful, but less harmful than smoking cigarettes’, ‘more harmful than smoking cigarettes’, ‘don't know’.  d‘Other perceptions’ include ‘not at all harmful’, ‘harmful, but less harmful than smoking cigarettes’, ‘as harmful as smoking cigarettes’, ‘don't know’.  e‘Other perceptions’ include ‘not at all harmful’, ‘harmful, but less harmful than smoking cigarettes’, ‘as harmful as smoking cigarettes’, ‘more harmful than smoking cigarettes’ | | | | | | | | | | | | | | | | |

| Supplementary table 3: Associations between harm perceptions of nicotine pouches and packaging condition, excluding participants who had never heard of or do not know what nicotine pouches are; Adults in the UK 2024 (N=2114)^a^ | | | | |
| --- | --- | --- | --- | --- |
|  | Harmful, but less harmful than smoking cigarettes (ref) | Other ^b^ | | |
|  | %(n) | %(n) | AOR(95%CI) | p |
| Branded packaging | 59.5(314) | 41.5(214) | 1.00 | Ref |
| Standardised packaging with usual descriptors | 63.2(323) | 36.8(188) | 0.84(0.65-1.09) | .179 |
| Standardised packaging with limited flavour descriptors | 59.8(308) | 40.2(207) | 1.01(0.78-1.30) | .951 |
| Standardised packaging with limited flavour and standardised nicotine descriptors | 61.2(343) | 38.8(217) | 0.96(0.75-1.24) | .772 |
| ^a^ Models are adjusted for smoking status, pouch use, age, gender, ethnicity and perceived financial status.  Due to small cell counts, ethnicity was collapsed into ‘White’ and ‘Racialised minorities’. Gender was also collapsed into ‘Female’ and ‘Other’  ^b^ ‘Other perceptions’ include: ‘not at all harmful’, ‘as harmful as smoking cigarettes’, ‘more harmful than smoking cigarettes’, ‘don't know’. | | | | |

Alt text: The graph shows the percentage of people who believed that the pouch image viewed was ‘not at all harmful’, 'harmful but less harmful than smoking cigarettes', ‘as harmful as smoking cigarettes’, ‘more harmful than smoking cigarettes’, ‘don't know’. The bars are highest for 'harmful but less harmful than smoking cigarettes' with around 55%-60% of people perceiving this. The bars are lowest for 'not at all harmful' with 2.6%-4% reporting this perception. There are separate bars for people who saw the different types of packaging, however there is very little difference between these groups. The darkest green bars represent people who saw branded packaging, the second darkest green bars represent people who saw standardised packaging with usual descriptions, the third darkest green bars represent people who saw standardised packaging with standardised flavour descriptions, and the lightest green bars represent people who saw standardised packaging with standardised flavour and nicotine descriptions.
